# Analyzing Antecedent Configurations of Group Emotion Generation in Public Emergencies: A Multi-Factor Coupling Approach

**DOI:** 10.3390/bs15010041

**Published:** 2025-01-03

**Authors:** Xiaohan Yan, Yi Liu, Yan Chen, Tiezhong Liu

**Affiliations:** School of Management, Beijing Institute of Technology, 5 Zhongguancun South Street, Beijing 100080, China; yxh0522@bit.edu.cn (X.Y.); liuyi120059745@163.com (Y.L.); chenyan@bit.edu.cn (Y.C.)

**Keywords:** group emotion, social combustion theory, fuzzy-set qualitative comparative analysis, generation pathways, multi-factor coupling

## Abstract

To enhance emergency management and public opinion governance, improve the accuracy of forecasting group emotional responses, and elucidate the complex pathways of multi-factor coupling in the formation of group emotions, this study constructs a theoretical framework grounded in the social combustion theory. Through web scraping and text sentiment analysis, group emotional tendencies were measured in 40 public emergency cases from the past five years. Using the fuzzy-set qualitative comparative analysis (fsQCA) method, the study explored the coupling, configuration effect, and formation pathways of factors such as “burning substance”, “accelerant”, and “ignition” in the emergence of group emotions. The results reveal significant differences in the generation pathways of positive versus negative group emotion. Inter-group threat as a “burning substance” is more likely to trigger negative group emotion, while “accelerant” plays a pivotal role in shaping and guiding emotional responses. Notably, the government’s response speed is critical for fostering positive emotions, whereas the emergence of rumors significantly contributes to the spread of negative group emotions. Additionally, the occurrence of stimulating events markedly increases the generation of negative group emotions. This study provides an important theoretical foundation and practical insights for the management and regulation of group emotions.

## 1. Introduction

With the widespread adoption of the Internet, the public increasingly relies on it for information, while diverse social platforms offer channels for expressing emotions and opinions. The development of the Internet and social platforms facilitates the dissemination of information related to public emergencies and the generation and evolution of emotions. The anonymity and interactive nature of the Internet exacerbate the emotional expression of users. Negative emotions accumulate and intensify within communities, leading to widespread societal negative emotions that may result in panic and chaos, potentially threatening social stability. This is particularly evident in accidents and disasters with significant casualties or events that reinforce public stereotypes where negative emotional responses are more pronounced. Without timely guidance and management, these emotions can escalate into secondary events, posing challenges to social governance. Therefore, it is crucial to explore the generation of group emotions in public emergencies.

Group emotions refer to the phenomenon where individual emotions influence each other and spread continuously under the stimulation of the external environment, resulting in a concentrated and emergent emotional state (Yao et al., 2022; Maitner et al., 2006). In this study, group emotions are defined as the relatively unified and strong emotions that emerge within a group of individuals following a sudden public event. Current classifications of public emotions are primarily divided into dichotomies (positive and negative) (He et al., 2017) and trichotomies (positive, negative, and neutral) (Cui et al., 2019). Most researchers classify group emotions using a binary approach, studying the interaction and stability between negative and positive emotions (Zhang & Xiao, 2023). Therefore, this study explores group emotions in public emergencies from the perspectives of positive and negative group emotions. Existing research studies on group emotions often focus on the spread or infection of emotion or use single cases to explore influencing factors. However, there is insufficient attention to the generation mechanism of emotions, and few studies investigate how combinations of different factors influence the generation of group emotions, which is a complex process. Single-case study methods lack the generalizability needed to identify consistent patterns, making it difficult to draw broader conclusions. In contrast, most existing group emotions studies tend to be quantitative single-case studies. For example, research on the spread of emotions in public emergencies often employs quantitative methods such as surveys, statistical analysis, or network analysis to measure the intensity, spread, and impact of emotions on a large scale. Quantitative research was used to analyze the factors influencing the spread of negative emotions among social media users in the case of COVID-19 (Lu & Hong, 2022); data on the aftermath of the 8.13 flash flood in the Longcaogou Scenic Area, Sichuan Province, China, were used to identify emotional contagion and conduct simulated interventions (Chu et al., 2024). However, fewer studies have adopted qualitative approaches or mixed-methods designs to investigate the underlying mechanisms of group emotion generation. This highlights a research gap that this study aims to address by combining qualitative and quantitative perspectives. The use of the fuzzy-set qualitative comparative analysis (fsQCA) method in multi-case studies offers a more effective approach for analyzing the generation of group emotions, addressing the limitations of single-case study methods and quantitative analysis. Consequently, this method has garnered increasing attention from scholars. This method, through the study of multiple cases, clarifies the combinatory relationships among condition variables and their impact on outcome variables. The research focuses on the synergistic interactions among condition variables, identifying how these variables combine and interact. By analyzing the coupling effects among influencing factors, this result provides more systematic and comprehensive guidance for emotion management and mitigation efforts. Current research has demonstrated that the applicability of the fsQCA method to network public opinion research (Xie & Duan, 2024; L. Zhang et al., 2024; Pappas & Woodside, 2021; Cai & Shen, 2023). For example, Li et al. studied the generative mechanism of public opinion in public emergencies from an information ecology perspective, identifying five explanatory variables: event information, release subject, information audience, information technology, and information environment. The researchers also proposed three effective conditional configurations (Li & Cao, 2020). Another study by Li et al. extracted seven factors, including the launch platform, event popularity, public attention, media coverage, government intervention, reversal time difference, and reversal attribution. They also conducted configuration and path analyses on the reversal strength of online public opinion (W. Li et al., 2022). This study intends to analyze 40 cases of public emergencies using fsQCA to identify the key factors contributing to the generation and fermentation of group emotions. The goal is to elucidate the combination relationship and specific action paths of influencing factors in the generation of group emotions during public emergencies, thereby paving the way for the future research on the generation mechanism of group emotions in such contexts. This study will provide a new perspective for the future research on the generation mechanism of group emotions and fill the gap in the existing research.

In contemporary society, public emergencies often lead to significant emotional fluctuations and strong public reactions. This study aims to utilize fsQCA to explore the influencing factors and specific pathways in the generation of group emotions during public emergencies. According to social combustion theory, the factors affecting group emotion generation are categorized into three dimensions: “burning substance”, “accelerant”, and “ignition”. Given the unique nature of group emotions, this study will also examine the relationships between group identity, intergroup threat, and relative deprivation, as well as the combined effects of these conditions on the generation of positive and negative group emotions. The goal is to provide an in-depth analysis of the mechanisms behind group emotions in public emergencies and offer recommendations for managing these emotions effectively.

## 2. Theoretical Basis

### 2.1. Social Combustion Theory

In 2001, Academician Niu extended the natural combustion process of substances to sociological research, proposing the social combustion theory (Niu, 2001). In nature, combustion is driven by three factors: the combustion substance, the accelerant, and the ignition. When applied to sociology, the emergence of certain social phenomenon can be likened to the combustion of matter. In this context, the “combustible substance” refers to the disharmony between humans and nature or the contradiction between individuals (Shan & Gao, 2010). False media reports, emotional guidance from opinion leaders, and the spread of rumors act as “accelerants”, leading to the public misperceptions of social events or the intensification of negative emotions (Zhao & Tao, 2023). Factors that seriously influence the trajectory of social phenomena, such as sensitive social issues, revelations by netizen, and other public opinion events, serve as the “ignition” in social phenomena (Zhao & Tao, 2023). This theory has been applied to various areas, including the emotional communication mechanism of online public opinion (Yin & Lin, 2022; Wen & Yin, 2024; Wang & Zhang, 2024), the generation mechanism of reverse-news public opinion (Peng & Cheng, 2023), the online emotional dissemination model (Wang et al., 2021), and the evolution model of online public opinion in emergencies (Tian et al., 2018), addressing several problems effectively. In the latest study, the scholars construct the DBUS (Divorced–Burning–Unburning–Stable) model (T. Chen et al., 2024). Therefore, using this theory to analyze the generation of group emotions is well founded and reasonable.

### 2.2. Intergroup Emotion Theory

The concept of intergroup emotion, initially proposed by Smith, emphasizes emotional phenomena at the group level (Smith, 1993). Building on emotion appraisal theory, social identity theory, and self-categorization theory, American social psychologist Mackie developed the Intergroup Emotions Theory (IET), offering a novel perspective for studying intergroup relations (Mackie et al., 2000). Intergroup emotion refers to the emotional experience shared by individuals when they identify as members of a specific group and view the group as part of their psychological self. Individuals form and understand intergroup relations through the generation of social identity. When self-categorization and social identity co-occur, individuals assess external events from the perspective of the group, based on comparison between the in-group and out-group. Intergroup emotions subsequently become an integral part of the emotional experience of group members and, once triggered, influence intergroup behavior. Unlike individual emotions, intergroup emotions are experienced by members of a group, directed either within or outside the group (Mackie et al., 2000). The main premises of intergroup emotion theory include the following: (1) individuals who identify with a social group will evaluate and interpret events in relation to that group; (2) group evaluations can elicit intergroup emotions directed at both in-groups and out-groups; and (3) these group emotions can influence behavior both in-groups and out-groups (Garcia-Prieto & Scherer, 2016). Long et al. found that the intergroup threat manipulation indirectly influences consumers’ intentions to purchase ingroup/outgroup products through increased anger and decreased hope (Long et al., 2023).

### 2.3. Influencing Factors of Group Emotion

Through a comprehensive literature research, it is evident that both domestic and international scholars have extensively researched the factors influencing group and social emotions during public emergencies. However, the majority of these studies are quantitative, with fewer qualitative studies or those combining both methodologies. While many are single-case studies, there are fewer multi-case studies, and although there is substantial research on influencing factors, there is limited investigation into the relationship between factors.

The key areas of focus in the research on influencing factors of group emotion include the following: (1) Social Identity. The generation of group emotion is closely related to social identity (Y. Chen et al., 2017; C. Liu et al., 2022; Wildschut et al., 2014). (2) Situational Context. The generation and expression of group emotions are often affected by the situational factors (He et al., 2017; Zheng, 2017; Pedrosa et al., 2020; S. Li et al., 2020; Yin et al., 2015). (3) Group Structure. The structure and size of a group affect the generation and expression of group emotions (Stellar et al., 2017; Zhu & Wang, 2020). (4) Individuals Factors. Factors such as individual personality, emotional state, and mental health also impact group emotions (Twenge et al., 2019; Yi et al., 2018).

In summary, the generation and expression of group emotions is a complex process influenced by numerous factors. Future studies should delve deeper into the relationships between these factors and explore the impact of group emotions on individuals and society.

## 3. Methodology

### 3.1. Case Selection

In QCA research, it is essential to ensure that the selected cases meet both criteria of similarity and diversity to achieve a balance between homogeneity and variation (Rihoux & Ragin, 2017). This principle guides the case selection process in this study. To achieve this balance, we primarily rely on cases drawn from the “Zhi Wei Shi Jian” database, supplemented by data from Baidu Index, Sina Weibo, and CNKI. The cases were selected based on the following key criteria: Typicality: The cases should represent common public emergency situations, offering insights that reflect broader trends. Diversity: We ensured that the selected cases covered a range of different types of public emergencies, including natural disasters, technological accidents, and health crises, to provide a comprehensive view of the issue. Certainty: Only cases with clear, well-documented events and outcomes were considered to ensure reliable analysis. Comprehensiveness: The selected cases needed to provide sufficient data and context for detailed analysis. In total, 40 cases were selected, with eight cases from each year spanning 2018 to 2022, ensuring a mix of temporal representation (see Table 1). The focus was on selecting high-impact cases that generated wide discussion as these are likely to provide more insightful and generalizable findings. To address potential biases, we acknowledge that using publicly available databases may favor more widely covered events. We mitigated this by cross-referencing multiple sources and ensuring geographic and emergency type diversity. Nonetheless, we recognize that the available data may influence the selection composition. By applying these criteria, we ensure the cases are representative, enhancing the study’s credibility while being transparent about the limitations.

### 3.2. Variable Selection and Assignment

Based on a comprehensive literature review, field research on the generation and influencing factors of group emotions, and an in-depth analysis of 40 cases of public emergencies, this study identifies eight influencing factors. These factors are categorized under the dimensions of “burning substance”, “accelerant”, and “ignition” according to the social combustion theory and are used to analyze the generation of group emotions in public emergencies.

#### 3.2.1. Burning Substances

The “burning substances” comprise three variables: relative deprivation, group identity, and intergroup threat. In public emergencies, group emotions emerge either due to perceived threats to their own interests or through empathy with the suffering of other groups. This interpersonal contradiction is described as the burning substance in the social combustion theory. For example, during an investigation about a community in the Fangshan District of Beijing on 29 May 2023, we discovered that the community enforced strict indoor restrictions in November 2022. When these controls were lifted in other communities, the residents of that community immediately felt a sense of inequality and gathered to demand similar treatment. Individual emotions can spread and become amplified among group members, leading to the formation of group emotions when a majority exhibits consistent feeling (Maitner et al., 2006). Thus, group emotions are a form of collective behavior, following similar mechanism for their generation and propagation (Du et al., 2023). Prior studies indicate that a strong sense of individual relative deprivation directly causes group emotions (S. Zhang et al., 2009), while group identification (Yin & Zhang, 2015) and intergroup threat (Zhang, 2013) are significant conditions for group behaviors. Consequently, group identification, intergroup threat, and relative deprivation are crucial in the generation of group emotions and behaviors (Yin & Tang, 2013; Xue et al., 2013). These variables, which are feelings experienced by individuals in relation to groups, are studied as burning substances in this research.

Community residents experience relative deprivation when they perceive themselves as disadvantage compared to others. This feeling arises from comparisons that emphasize the “relative quantity” of resources (Stouffer, 1949; Callan et al., 2015). Scholars assert that individuals have inherent value expectations, and when social or class changes obstruct these expectations, a sense of relative deprivation emerges (Feierabend & Gurr, 1970). At the group level, this involves perceiving one’s group as disadvantage compared to others based on specific comparison principles (De la Sablonnire et al., 2015). Relative deprivation is a psychological process involving cognition and emotion. Individuals compare themselves to a reference group, perceive an inferior status, and generate negative cognitions and emotions, such as dissatisfaction, anger, jealousy, or resentment. This impacts mental health, physical health, and interpersonal or group relationships (Qin et al., 2022; Lyu & Sun, 2020; Ding et al., 2023). A strong sense of relative deprivation also influences intergroup dynamics and the formation of group emotions. In this study, the presence of relative deprivation was determined by applying a set of criteria, such as the following: Income Inequality: Whether there is a heated discussion due to significant differences in income or wealth; Social Comparison: Discussions in which negative comparisons between two groups arise (for example, when one community believes the other group has better living conditions or easier access to resources); Perceived Social Mobility: Reduced or perceived lack of opportunities for upward social mobility. To ensure consistency and reduce researcher bias, more than a dozen experts measured the presence or absence of relative deprivation at events against these criteria. If at least one of the above indicators occurs, “relative deprivation” (assigned a value of 1) is considered to be present in the event. If these conditions are not met, the value is assigned to 0. This structured approach reduces the possibility of subjectivity by relying on standardized survey measures, helping to mitigate bias and improve the reliability of the coding process.

Group identity refers to an individual’s sense of belonging to a particular group, believing they are members of this group and have a close relationship with it (Ellemers & Haslam, 2012). When group identity is established, individuals internalize the views, values, and norms of behavior recognized by group members into their own cognition (Sani, 2012). This internalization links individuals to other social members and encourages collective efforts to achieve group goals (Cruwys et al., 2014; Jetten et al., 2014). Consequently, group members tend to maintain consistent views and opinions. Group emotions differ from individual emotions. When individual strongly identify with a group, they may experience group emotions even if specific group events do not directly involve them (Smith et al., 2007). The relationship between group identification and negative group emotions is not conclusive. For instance, Dumon et al. found that higher group identification among white individuals correlated with lower group guilt over hurtful injustices by white South Africans against black people (Dumont & Waldzus, 2014). Conversely, Jelic et al. reported that group identification can significantly predict higher group guilt (Jelic et al., 2013). Given these findings, this study examines group identification as an influencing factor of group emotions. It aims to verify the role of group identification in the generation of group emotions and uses the presence of group identification as the measurement standard. Cases with group identification are assigned a value of 0, while those without it are assigned a value of 1.

Intergroup threat occurs when one group perceives its existence, development, or goal pursuit as threatened by another group. This threat can be either real or perceived by group members. Individuals experiencing intergroup threats are more likely to have negative emotions, such as shame, guilt, shame (Y. Chen et al., 2020), fear, vulnerability, anger, resentment, anti-empathy, schadenfreude and jealousy (Cikara et al., 2014). The perception of intergroup threat among group members triggers consistent group emotions. Studies have shown that various dimensions of intergroup threat, such as realistic threat, identity threat, and cultural threat, can affect group emotions and subsequently group attitudes. Therefore, this study considers the intergroup threat as the antecedent variable and uses the group’s perception of intergroup threat as measurement standard. Cases where the group perceives an intergroup threat are assigned a value of 1, while cases where the group does not perceive such a threat are assigned a value of 0.

#### 3.2.2. Accelerant

The “accelerant” category includes four variables: government intervention, guidance by opinion leaders, the help of the media, and rumors. Previous studies have examined the role of these four aspects—government, media, opinion leaders, and rumors—in influencing public opinion (W. Li et al., 2022; Peng & Cheng, 2023). Therefore, this study considers the communication and information dissemination by the government, media, opinion leaders, and rumors as “accelerants”.

Government response time refers to the guidance and measures taken by the government after public emergencies, such as responses to natural disasters, epidemic prevention and control, and the time required for official statements to guide public opinion. The government plays a leading role in managing online public opinion during public emergencies, significantly impacting the tendency and intensity of group emotions. Chen et al. argue that the intensity of external intervention information is crucial in shaping public opinion trends (T. Chen et al., 2020). As the primary entity announcing information during public emergencies, the government’s interaction with the public directly affects public emotions. Therefore, the government needs to respond in a timely manner to meet the public’s information needs and thus affect the trend of emotions (Y. Chen et al., 2022). Timely and effective intervention can take control of public opinion early, alleviate intensified conflicts, and promote positive development of public emotions during emergencies, preventing the spread of negative emotions. Therefore, this study includes the government response speed as a conditional variable, using the time required for an official response after public emergencies to measure government response speed.

The significance of opinion leaders in shaping online public opinion has been widely recognized by scholars (Park, 2013; Wan et al., 2022; Jain et al., 2020). Opinion leaders, including media personnel, experts, and network influencers with extensive knowledge, skills, or social influence, possess high reputation, discourse power, and the ability to incite and influence (Aven, 2014). Guidance by opinion leaders refers to the influence these individuals exert on public opinion during emergencies by releasing relevant statements. In such situations, the opinions and emotional tone of opinion leaders are crucial in controlling online public opinion and directing online emotions. Their views can significantly impact general social media users, thereby influencing the overall trend of public opinion and the effectiveness of public emergency management (Chen & Chen, 2015). Therefore, this study includes the guidance of opinion leaders as a conditional variable, measured by the number, influence, and volume of publications by opinion leaders involved in the discussing public emergencies and expressing emotional or inciting views. The specific calculation method is presented in Formula (1).

(1) OL=∑Infuence of opinions×Post number

$$Infuence\;of\;opinions=\left\{\begin{array}{c} 0;\;0\leq \mathrm{fans}\leq 10\;\mathrm{million}\\ 1;\;10\;\mathrm{million}\leq \mathrm{fans}\leq 20\;\mathrm{million}\\ 2;\;20\;\mathrm{million}\leq \mathrm{fans}\leq 30\;\mathrm{million}\\ 3;\;30\;\mathrm{million}\leq \mathrm{fans}\leq 40\;\mathrm{million}\\ 4;\;40\;\mathrm{million}\leq \mathrm{fans}\leq 50\;\mathrm{million}\\ 5;\;\mathrm{fans}\geq 50\;\mathrm{million} \end{array}\right.$$

The help of the media refers to the role of mainstream media in actively guiding public opinion and setting the agenda during public emergencies. The media speaks for the interests of various groups and classes, responds to the public doubts and expectations, conveys accurate news, and publicizes government response measures promptly. Effective communication with the public helps understanding their thoughts and demands, guiding the direction of public opinions, and channeling negative emotions. Media participation can increase the attention from the public, government, and other relevant departments to the event, acting as a catalyst for the development and emotional trajectory of the situation (W. Li et al., 2022; Peng & Cheng, 2023). The volume of news reports and the extent of media participation are critical in driving online public opinion (Li & Li, 2019). Therefore, this study includes the help of the media as a conditional variable influencing group emotions, measured by the number of media outlets engaging in appropriate agenda setting and positive guidance.

Existing studies have extensively analyzed the relationship between rumors and emotions (Z. Liu et al., 2023; Wang & Qiu, 2021). In this study, most selected cases involved rumors while aligning with the definition of irritant events in “ignition”. Therefore, rumors are chosen as the “ignition” variable. During public emergencies, various rumors and false information often arise, leading to difficulties and dilemmas as they to ferment and spread, exacerbating the situation. The presence of rumors frequently results in heightened negative emotions, such as fear, anger, and distrust, which can further escalate the crisis (Ji et al., 2014; Dong & Qi, 2023). Hence, the occurrence of rumors can be regarded as the “ignition” that triggers group emotions in public emergencies. In this study, the presence of rumors is treated as a conditional variable influencing group emotions. To reduce subjective bias in coding, we developed a set of objective criteria for identifying cases involving rumors. The presence of rumors is determined based on the following measurable indicators: Media Reports: Articles or news reports that explicitly mention rumors or false information circulating during the event; Social Media Posts: A significant volume of social media discussions or shares regarding false or unverified information; Public Statements from Authorities or Experts: Any official denial or clarification issued in response to misinformation. If at least one of these indicators is met, a case is assigned a value of 1 (indicating the presence of rumors). If none of these indicators are present, the case is assigned a value of 0 (indicating the absence of rumors). This approach ensures that the identification of rumors is based on observable and verifiable data, reducing the potential for researcher bias and increasing the reliability of the results.

#### 3.2.3. Ignition

“Ignition” refers to social hot spots, significant events, and other irritant events, and, for group emotions in public emergencies, it refers to the fuse factor that directly triggers group emotions (Shan & Gao, 2010). When an irritant event occurs, it captures the attention of netizens and can even lead to collective public actions, resulting in the generation of group emotions. Such an event can be referred to as a “tipping point”. Malcolm illustrates how to identify tipping point by analyzing the three laws involved in the emergence of popular trends: the law of the few, the stickiness factor, and the power of context. Zhang et al. (Zhang & Chen, 2012) examined the triggering effect of irritant events on the emergence of online mass incidents, while Gao et al. (Gao & Hao, 2020) analyzed how stimulating events awaken collective memory and subsequently driven the spread of public opinion, fostering a unified emotional response and emotional mobilization. In this study, irritant events are treated as a condition variable for group emotion, with their presence or absence serving as the measurement criterion. Case variables involving irritant events are assigned a value of 1, while those without such events are assigned a value of 0.

**Figure 1:**
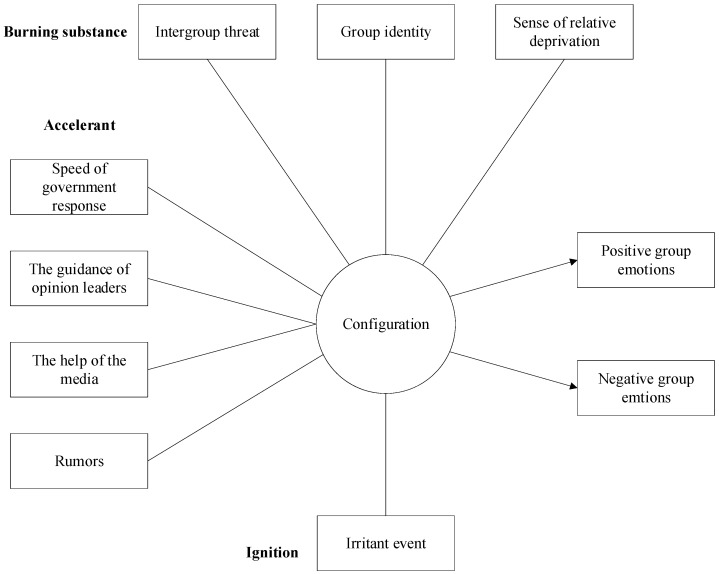
Analytical framework of group emotion generation for public emergencies.

This study examines the generation mechanism of group emotions during public emergencies, using the emotional tendencies in related texts as an index. Group emotions, the outcome variable, are measured by the proportion of positive and negative emotional expressions in posts and comments on platforms like Weibo. Data collection began with identifying keywords and hashtags linked to public emergencies. Using Python scripts, posts and comments from official accounts, media outlets, and general users were scraped within specific time windows to capture immediate public sentiment. Sentiment analysis was conducted with ROSTCM6, a Chinese text mining tool, which classified texts as positive, negative, or neutral based on emotional tone. The analysis process included the following: Text Preprocessing: Cleaning raw data by removing irrelevant content (e.g., URLs, special characters, and stop words). Sentiment Classification: Categorizing texts using a machine learning model trained on Chinese texts. Emotion Proportion Calculation: Calculating the proportions of positive and negative emotions, used as dependent variables to analyze group emotions’ intensity and fluctuations. This approach provides a framework for understanding group emotions during public emergencies, as depicted in Figure 1.

In order to ensure the credibility of the case coding assignment, 13 social emotion research experts cooperated in the coding work of the case according to the specific situation and the above coding principles. The experts then coded 40 cases independently and discussed any differences in their tasks. The specific criteria for assigning variables are shown in Table 2.

### 3.3. FsQCA Method

Fuzzy-set qualitative comparative analysis (fsQCA) is a research method that integrates quantitative and qualitative methods based on Boolean algebra. It addresses complex causal relationship from a holistic perspective and can identify various reasonable configuration conditions (Yi et al., 2018). Three common operational methods are csQCA, fsQCA, and msQCA, with csQCA and fsQCA being more widely used. Further, fsQCA offers two types of configurations that include necessary and sufficient conditions. These configurations may be marked by their presence, their absence, or a “do not care” condition. The necessary and the sufficient conditions lead to a distinction between core and peripheral elements. Core elements are the ones with a strong causal condition with the outcome; peripheral elements are those with a weaker one (Fiss, 2011). The covariance that exists among the variables in a model indicates that the presence or absence of one variable may influence their effect, both on the other variables and on the outcome, supporting the need to apply configurational analysis, which is based on this notion (Fiss, 2007). Building on the existing research, this study collected data on 40 public emergency cases to explore the pathways and mechanisms of group emotion generation using eight typical antecedent variables. To calculate the group emotions associated with these emergencies, web crawling technology was employed to gather Weibo posts during the peak period of the events. The collected posts were then analyzed using ROSTCM6 software for text sentiment analysis.

### 3.4. Data Calibration

Before conducting fsQCA analysis, it is necessary to calibrate the data by standardizing it with anchor points so that the values fall within the range of 0–1. The three anchor points used are full affiliated points, intersection points, and completely unaffiliated points. In this study, the 95th percentile, 50th percentile, and 5th percentile of the variables were used as full affiliated, intersection, and completely unaffiliated points, respectively, for direct calibration. Variables such as government response speed, guidance by opinion leaders, the help of the media, positive group emotions, and negative group emotions were calibrated accordingly. The specific calibration anchors are shown in Table 3. To ensure data with a membership degree of 0.5 is included in the truth table analysis, it is adjusted to 0.501 by adding 0.001, following Fiss’s methodology (Fiss, 2011).

## 4. FsQCA Findings

### 4.1. Single-Factor Necessity Analysis

First, we conducted a necessity test on the conditional variables that influence the generation of group emotions to determine whether each antecedent variable is a necessary condition for a specific outcome. Necessity, from a set theory approach, means that a condition is a superset of the outcome (Ragin & Fiss, 2008); thus, for each case in the sample, the fuzzy-set membership score of the outcome is smaller than the fuzzy-set membership score of the causal conditions. For a condition to be necessary, its consistency should exceed the threshold of 0.9 (Schneider & Wagemann, 2010). Consistency is the degree to which the cases in the sample that share a causal condition or configuration agree in displaying the focal outcome (Ragin & Fiss, 2008).

**Figure 2:**
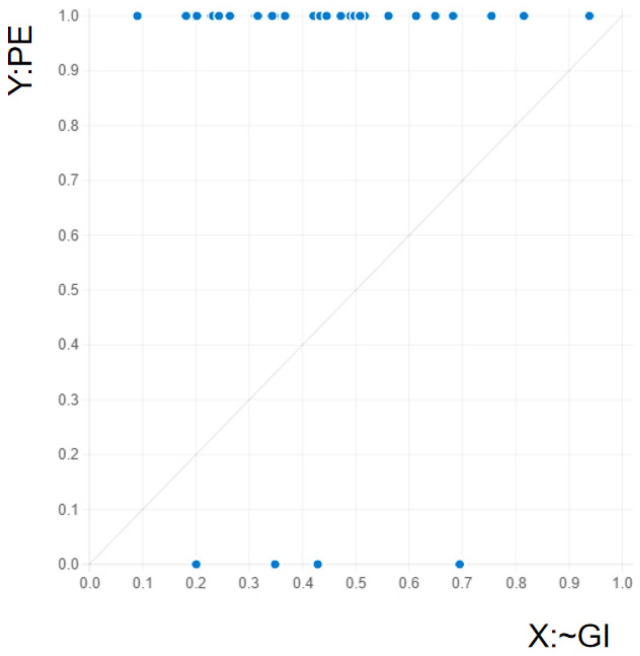
Fuzzy XY plots for testing necessary condition.

The findings from the analysis of necessity are presented in Table 4. We test for necessary conditions both for positive group emotions and negative group emotions. In detail, for positive group emotions, the consistency values range between 0.0964 and 0.9036 for both the presence and negation of the causal conditions. The consistency of non-group identification is more than 0.9, indicating that non-group identification may be a necessary condition for generating positive group emotions (Schneider & Wagemann, 2010), which is considered to be a possible necessary condition for generating positive group emotions. However, the coverage score for this condition is less than 0.5. This indicates that it is not a necessary condition (Ragin, 2010), and the XY graph shown in Figure 2 verifies this conclusion. Therefore, we proceed to the test of sufficient conditions. On the other hand, for negative group emotions, none of the causal conditions exceeds the value of 0.9. Thus, we proceed with the fuzzy-set analysis to identify sufficient combinations of causal conditions that explain negative group emotions.

### 4.2. Conditional Configuration Analysis

Configuration analysis aims to examine the adequacy of different configurations in explaining the resulting variables. Each configuration represents different conditions leading to the same outcome. In this context, a “solution” refers to a combination of conditions consistently supported by a sufficient number of cases, validating the rule that “the combination leads to the outcome”. After calibration, the fsQCA (version 4.1) software generates a truth table comprising 2^k^ rows (k being the number of predictors), which represents all possible combinations of conditions along with their respective frequencies (i.e., the number of observations for each combination). Many combinations may exhibit zero frequency. The table also reports consistency, which measures how well the observed cases align with the set-theoretic relationship (Fiss, 2011; Ragin & Fiss, 2008). Researchers typically sort the truth table by frequency and consistency. A frequency threshold is then set to ensure a minimum number of observations per combination. For large samples, the threshold is typically set at 3, while for smaller samples (<100 cases), it is set at 2 (Fiss, 2011; Ragin & Fiss, 2008). For small and medium-sized samples, a threshold of 1 may suffice, but for larger samples (e.g., 150 or more cases), a higher threshold is recommended (Ragin & Fiss, 2008). In this study, the threshold is set at 1, excluding combinations with lower frequencies. Additionally, a consistency threshold must be established, typically above the recommended value of 0.75 (Ragin & Fiss, 2008). A lower consistency threshold helps to identify more necessary conditions, reducing type II errors (false negatives) at the cost of increasing type I errors (false positives), and vice versa (Dul, 2010). In this study, the consistency threshold is set at 0.8 (Huang & Yang, 2023), ensuring that over 80% of the cases are included in the final analysis (Ragin & Fiss, 2008; Schneider & Wagemann, 2012). Combinations exceeding this threshold are deemed sufficient to fully explain the outcome, with their outcome values set to 1, while the remaining combinations are assigned a value of 0.

FsQCA provides three solution types: complex, parsimonious, and intermediate solution. The complex solution includes all combinations, while the parsimonious and intermediate solutions simplify the interpretation (MacKenzie & Podsakoff, 2012). The intermediate solution, which also includes the parsimonious solution, identifies core conditions (present in the parsimonious solution) and peripheral conditions (appearing only in the intermediate solution) (Fiss, 2011). Therefore, the analysis is primarily based on the intermediate solution, with core and auxiliary conditions distinguished by referring to the simple solution. Conditions present in both the intermediate and reduced solutions are the core conditions, while those only appearing in the intermediate solution are considered auxiliary conditions. 

(1) Configuration analysis of positive group emotion

Conditions (core or peripheral) may be either present, negated, or absent with no influence on the solution. Consistency values are presented in Table 5 for each solution as well as the overall solution, with all values being higher than the recommended threshold (>0.75) (Bi & Cao, 2021). Consistency shows the degree that a relationship has been approximated, and coverage evaluates the empirical relevance of a consistent subset (Ragin, 2006). The overall consistency is similar to the correlation showing how strong is the solution, and the overall solution coverage indicates the extent to which positive group emotions may be determined from the existing configurations. The overall solution coverage of 0.2720 shows that about 27% of the outcome is explained by the four solutions (Xu et al., 2020). Furthermore, fsQCA computes the empirical relevance for each solution by calculating raw and unique coverage. The raw coverage describes the amount of the outcome that is explained by a certain alternative solution, while the unique coverage describes the amount of the outcome that is exclusively explained by a certain alternative solution. The findings from the fsQCA on the configurations for positive group emotions are presented in Table 6. Every combination in the solution is able to explain the same outcome at a specific amount. The solutions presented in Table 6 explain a great number of positive group emotions, ranging from 3.75% to 11.98% cases associated with the outcome.

The speed of government response is a core determinant of positive group emotion. In the four configurations, a rapid government response is a common factor. This finding suggests that, during public emergencies, timely government actions and transparent information dissemination significantly influence the trajectory of public emotions, particularly when there is a perceived sense of relative deprivation (as seen in H1a and H1c). The central importance of response speed highlights that an efficient government reaction can more effectively evoke positive group emotions. For instance, during natural disasters or social unrest, such as the “A 6.0-magnitude earthquake struck Changning in Yibin” and the “Extremely heavy rainstorm in Henan,” prompt governmental intervention and guidance foster a sense of security and unity among the public, thereby encouraging positive emotional responses.

A sense of relative deprivation serves as a key factor in generating positive group emotions. In configurations H1a and H1c, public experiences of relative deprivation and inter-group threat were mitigated by proactive government responses, fostering positive emotions. Cases such as “A female passenger is dragged by security guards on the Xi’an subway” and “Abundance nest express cabinet overtime charge controversy” highlight that minor public emergencies, when resolved effectively, can lead to favorable emotions despite the presence of rumors or irritant events. In configurations H1b and H1d, the absence of relative deprivation and rumors, coupled with timely government information disclosure, led to positive emotions regardless of perceived threats or group belonging. Incidents like the “Sichuan Airlines diversion” and “Dr. Li Wenliang, the whistleblower of the epidemic, has died of COVID-19” featured unexpected events, heroic acts, and transparent communication, which shifted public focus to concern for those affected and admiration for the heroes, fostering positive group emotions.

(2) Configuration analysis of negative group emotion

Consistency values are presented in Table 7 for each solution as well as the overall solution, with all values being higher than the recommended threshold (>0.75) (Bi & Cao, 2021). The overall solution coverage is 0.6295, which indicates that the model has strong explanatory power (Xu et al., 2020). The findings from the fsQCA on the configurations for negative group emotions are presented in Table 8. The solutions presented in Table 8 explain a great number of positive group emotions, ranging from 4.91% to 30.28% cases associated with the outcome. The large number of pathways highlights the complex social–psychological mechanism underlying negative group emotions. In analyzing these eight paths, group identity, the presence of rumors, and the occurrence of triggering events appear most frequently.

The formation of negative group emotion is shaped by multiple factors, including inter-group threats, rumors, and the role of the media. Intergroup threats are commonly found as core conditions, indicating that the antagonistic relationships between internal and external groups can intensify negative emotions, particularly during periods of social unrest or crisis. For example, incidents such as “A woman takes a Didi Hitch ride in Yueqing and is killed” and “A group of men beat up a girl at a barbecue restaurant in Tangshan” have prompted vulnerable groups, such as women, to reflect on the perceived threats to their collective interests, consequently leading to heightened negative group emotions.

Rumors play a crucial role in generating negative group emotions. Their frequent appearance across multiple paths indicates that the loss of control and uncertainty in information dissemination are significant drivers of negative emotions. This highlights the importance of constructing and managing an effective information environment to channel negative group emotions more constructively. In configurations H2b and H2d, rumors are core conditions, demonstrating that whether through the collective efforts of government, opinion leaders, and the media (H2d) or the independent role of the media (H2d), negative emotions are still generated. In these cases, rumors, to a varying degree, attracted more public attention and incited stronger negative emotions like anger compared to objective facts. Rumors foster public perceptions of relative deprivation, a heightened sense of belonging, or intergroup threats, rendering media, opinion leaders, and government largely ineffective in guiding positive group emotions. In configurations H2c, H2f, and H2g, rumors are present as marginal conditions, along with factors such as group identify, relative deprivation, or intergroup threat, contributing to negative group emotions. In configurations H2e and H2f, while rumors are absent, a clear sense of group belonging and perceived group threat remain. The limited involvement of opinion leader in these cases results in strong negative emotions. Such incidents, often characterized by significant casualties and impact, are typically accidents or disasters with unclear causes. The absence of timely and effective guidance from media, opinion leaders, and government amplifies public anger and other negative emotions.

Irritant events act as an ignition point, playing a critical role in triggering negative group emotions. These events are featured in multiple pathways leading to negative emotions (H2a, H2c, H2d, H2f, and H2h), indicating their capacity to evoke public negative emotions such as fear, anxiety, and anger. Sudden and unpredictable incidents, whether natural disasters or social events, can cause emotional volatility and a loss of control among group members. For example, Zhai Tianlin’s dismissive comment on Weibo stating he was “just joking”, coupled with his studio’s claim that the paper was uploaded with university approval, sparked widespread dissatisfaction. The interaction between intergroup threats and irritant events can amplify the intensity of negative emotions. When faced with intergroup threats, the public’s perception of external dangers heightens, exacerbating fear and anxiety. The government’s response speed to irritant events is a crucial determinant in managing public emotions. A swift and effective response can mitigate the escalation of negative sentiment, whereas a delayed response can exacerbate public discontent and anger.

In addition, in some cases, public stereotypes are stimulated, leading to negative emotions. These events triggered stereotypes about fairness and evoked past emotions regarding criminal activity and epidemic prevention. Without the intervention of opinion leaders and the government, the media alone struggled to channel these negative emotions effectively.

### 4.3. Robustness Test

To ensure the robustness of the study, we tested the analysis by altering the threshold parameters. First, we adjusted the consistency threshold from 0.8 to 0.85, keeping all other steps unchanged. This adjustment showed that the configuration path and core conditions remained consistent with the original study, and the consistency and coverage metrics were unaffected. Secondly, we tested robustness by modifying the data calibration points based on relevant research results. Specifically, we adjusted the original anchor points to quartile points, while keeping other steps unchanged. Further analysis revealed that the configuration paths obtained when positive group emotion was used as the outcome variable align with the original configuration paths. Similarly, the configuration paths derived when negative group emotion was used as the outcome variable also match the original configuration paths. These findings indicate that the configurations identified in this study possess a degree of robustness.

## 5. Discussion

Based on social combustion theory, this study utilizes fuzzy-set qualitative comparative analysis (fsQCA) to explore the mechanism behind group emotion generation in public emergencies, focusing on the impact of the three dimensions—“burning substance”, “accelerant”, and “ignition”—on the development of group emotions. Additionally, the study examines the mechanism by which different combinations of variables contribute to group emotions generation. Through necessity analysis and configuration path analysis for both positive and negative group emotions in public emergencies, the main conclusions of this study are as follows:

The pathways to positive and negative group emotions in public emergencies differ significantly. Configuration analysis reveals four pathways to positive emotions and eight to negative emotions. These findings indicate that group emotions arise through various pathways, requiring the combined influence of multiple factors. Positive and negative emotions are not simply opposites; they represent distinct processes with different generative pathways. From an evolutionary perspective, positive and negative emotions serve different adaptive functions (Xu et al., 2020). Negative emotions, which evolved to promote survival in life-threatening situations, trigger physiological responses that help individuals react to immediate dangers (Yang et al., 2007). In contrast, positive emotions, as explained by the “broaden-and-build theory”, enhance attention, cognition, and behavior, providing long-term adaptive benefits (Fredrickson, 2001, 2003; Fredrickson & Branigan, 2005). At the group level, negative emotions often act as collective defense mechanisms in response to perceived threats, fostering cooperation. Positive emotions, on the other hand, promote collective growth and shared progress.

The “burning substance” is a key element in the generation of group emotions. For instance, relative deprivation can foster a strong sense of community, encouraging individuals to unite and generate positive emotions. However, it can also lead to dissatisfaction and frustration, triggering anger or anxiety, which fuels negative emotions. A strong group identity enhances positive emotions, particularly in the face of external threats, but has minimal direct impact on negative emotions. This may be because, in situations of intergroup conflict, strong identity can increase internal tensions and exacerbate negative emotions. While intergroup threats can unify groups in certain conditions, they are primarily catalysts for negative emotions, amplifying fear and anxiety, especially in competitive or conflict-driven environments. When relative deprivation and group identity combine, they can strengthen group cohesion, but in the presence of intergroup threats, this combination may heighten negative emotions.

Key accelerants, such as timely government intervention, guidance from opinion leaders, media amplification, and the spread of rumors, are crucial in shaping group emotions. Government responses are particularly important—prompt, accurate information from authorities can steer the public toward positive emotions. The interaction between “burning substance” and “accelerants” is more likely to generate positive group emotions, while the combination of “burning substance” and “ignition” tends to foster negative emotions. Research supports the role of media, government, and opinion leaders as pivotal agents in online public opinion guidance and governance. This study’s findings align with previous work, showing that a swift, effective government response enhances public trust and fosters positive emotions, especially during crises. Conversely, delayed or inadequate responses can fuel dissatisfaction and anger, amplifying negative emotions. Opinion leaders who spread positive information can encourage constructive actions, while those who incite confrontation exacerbate negative emotions, particularly in times of social unrest. Media coverage also plays a significant role: positive reports can strengthen collective identity and promote positive emotions, whereas negative or conflict-focused reporting tends to amplify fear and anxiety. Rumors, though sometimes used to rally group sentiment, typically spread fear and insecurity, further intensifying negative emotions, especially in crises with information asymmetry. Studies show that false information, like rumors, can create and amplify public opinion risks. In public emergencies, rumors often resonate with societal stereotypes, such as resentment toward wealth or power, fueling social discontent. This makes the spread of rumors a potent accelerant for negative group emotions. Opinion leaders, often collaborating with media, shape public sentiment, and, under the influence of rumors, this dynamic can quickly escalate negative emotions.

The presence of an “ignition” factor is more likely to lead to the emergence of negative emotions. Irritant events, in particular, can evoke a sense of relative deprivation, foster strong group identity, and heighten perceived group threat, thereby triggering negative emotions. While some irritant events can ignite positive group emotions, motivating group participation and action, sudden conflicts often induce panic and anxiety, accelerating the onset of negative emotions. When such events occur, they activate the interaction between “burning substances” and “accelerants”, leading to rapid shifts in collective emotions. This dynamic underscores the importance of managing responses to stimulating events to prevent the escalation of negative group emotions.

“Burning substances” provide the foundation for the emergence of group emotions, while accelerant influence the spread and development of these emotions, either accelerating or decelerating them. The “ignition” acts as the critical factor that triggers emotional responses. When relative deprivation and group identity are pronounced, positive group emotion can be stimulated, especially in the absence of external threat. However, there emotions are more likely to change into negative group emotion when intergroup threats are present. The speed of government response can mitigate the negative effects of relative deprivation and help to guide group emotions in a positive direction. The influence of opinion leaders depends on the nature of the content they disseminate. The combination of media coverage and rumors often amplifies negative group emotions, while irritant events act as catalysts that activate different combinations, resulting in rapid changes in group mood.

This study has both theoretical significance and practical implications. Theoretically, it applies social combustion theory to the field of group emotion research, offering an in-depth analysis of the complex mechanism underlying group emotion generation. Social combustion theory, which originally focused on the dynamics of collective action and group behavior, is adapted here to explore how emotional states propagate and intensify within a group during public emergencies. By examining the ways in which emotions spread, amplify, and transform within groups, this study enhances the theoretical understanding of group emotion dynamics. The application of this theory to group emotions broadens its scope, offering a new framework for studying both negative and positive emotional responses in groups. It shifts the focus from individual emotional triggers to the interaction and interdependence of various factors that contribute to collective emotional states. Furthermore, this study uses the fsQCA method to identify multiple pathways in the generation of group emotions, expanding its use in this context. While previous research has predominantly focused on individual factors, this approach emphasizes the interplay of these factors and offers a more holistic understanding of the processes at work. In addition, by integrating the concept of relative deprivation—identified through field investigations—into the theoretical framework, this study provides a more nuanced and comprehensive perspective on the formation of group emotions. The incorporation of relative deprivation highlights the role of perceived inequalities in emotional dynamics, further enriching the understanding of how group emotions emerge and evolve. Moreover, this study bridges the gap between qualitative and quantitative approaches by systematically analyzing 40 public emergency cases. By combining case study analysis with quantitative methods, it offers a more robust understanding of group emotions than previous research, which often relied on either qualitative or quantitative methods in isolation. This multi-case design enriches the study by providing a richer, more diversified empirical foundation for understanding the complex dynamics of group emotions during public emergencies.

In terms of practical implications, the findings of this study offer practical guidance for managing group emotions during public emergencies. When emergencies trigger stereotypical thinking and reinforce biases, government responses alone may be insufficient. Active involvement from opinion leaders and the media is crucial for helping the public form objective, rational judgments, steering them away from emotionally charged narratives such as “hatred for the rich” or “hatred for officials.” This approach prevents emotional extremes and aligns with social combustion theory, which highlights the role of social dynamics and influencers in shaping collective emotions. By leveraging these dynamics, authorities can better manage public sentiment and promote balanced emotional responses. In cases where the cause of an emergency is unclear, encouraging the public to view events objectively, rather than over-identifying with affected parties, can help to guide group emotions in a positive direction. Media and opinion leaders can play a pivotal role in disseminating accurate, rational information to reduce collective distress. This suggests that integrating social combustion theory with crisis communication strategies could improve emotional management in emergencies. When public emergencies have limited direct impact but high potential for inciting conflict, clarifying facts quickly is essential to defuse tension and prevent emotional confrontations. Neutral opinion leaders, avoiding unnecessary incitement, can help to prevent negative emotional escalation. This underscores the practical value of social combustion theory in crisis management and opens new avenues for research on information control and emotional guidance in conflict prevention. Finally, managing rumors is crucial to emotional guidance efforts. Preparedness for rumor-prone incidents, transparency in communication, and the timely refutation of rumors are key to mitigating negative group emotions. Future research could explore the dynamics of rumor propagation and its impact on emotional contagion, particularly through the lens of social combustion theory. Research could also investigate how these strategies can be systematically applied across different types of public emergencies, focusing on the interplay between media, opinion leaders, and group identity to promote positive emotional outcomes. Additionally, the role of social media in amplifying group emotions offers another promising area of study. Integrating these findings with existing theoretical frameworks, such as social combustion theory, could lead to more refined models of group emotion dynamics and offer practical insights for crisis management.

## 6. Conclusions

This study constructs a comprehensive framework for understanding the factors influencing group emotion generation in public emergencies, drawing on social combustion theory, prior research, and case analysis. Categorized into the dimensions of “burning substance”, “accelerant”, and “ignition”, the framework is examined using fuzzy-set qualitative comparative analysis (fsQCA) and text sentiment analysis. The findings identify four pathways for positive group emotions and eight for negative group emotions, highlighting the configuration effects of variable interactions. Key insights reveal that government response speed is a core determinant of group emotions, with the presence of “ignition” significantly increasing the likelihood of negative group emotion outbreaks. Rumors further amplify this effect. Importantly, the study uncovers distinct pathways for the generation of positive and negative group emotions, offering nuanced perspectives on their dynamics. These findings provide valuable guidance for effectively managing and channeling group emotions during public emergencies.

Despite these contributions, several limitations warrant attention. First, the variables representing “burning substance”, “accelerant”, and “ignition” were not fully explored in the study. Future research should expand the range of variables, incorporating factors such as the type of issue at hand, which could act as a key driver of group emotion generation. Additionally, the assignment process for various variables, particularly in text sentiment analysis, presents challenges in accuracy and interpretation, suggesting that refining this process could improve the reliability of results. A key limitation of the fsQCA method, as applied in this study, is its sensitivity to sample size. While the 40 cases analyzed are generally considered sufficient for fsQCA, smaller or larger sample sizes may yield different results, thus affecting the robustness and generalizability of the findings. Future research should explore how varying sample sizes impact the stability of configurations and the interpretability of results. Moreover, the fsQCA method faces constraints with an increasing number of variables. The exponential growth of possible configurations with additional variables can complicate interpretation. In this study, we limited the number of antecedent variables to eight to maintain manageable analysis, but future research may need to employ dimensionality reduction techniques or alternative approaches to handle more complex configurations. In light of these limitations, future studies could focus on refining the fsQCA method by exploring alternative techniques for handling larger datasets and more variables. Additionally, future research may benefit from investigating the effects of sample size variation on fsQCA outcomes, as well as incorporating a broader set of influencing factors into the study of group emotions. This would allow for a more nuanced and comprehensive understanding of the dynamics of group emotions in public emergencies.

## Figures and Tables

**Table 1:** Typical cases of public emergencies.

ID	Name	Time	ID	Name	Time
1	Sichuan Airlines diversion	14 May 2018	21	Bao Yuming, an executive at a listed company, is suspected of sexually assaulting his adopted daughter	8 April 2020
2	Qingyang girl jumping from building	20 June 2018	22	Abundance nest express cabinet overtime charge controversy	27 March 2020
3	Changsheng and other vaccine fraud	15 July 2018	23	“Fake milk powder” has led to the emergence of big-head dolls in Hunan	12 May 2020
4	Shouguang flood discharge	19 August 2018	24	A woman in Hangzhou mysteriously disappeared late at night	18 July 2020
5	On G334 high-speed train, a man pretending to be sick to obtain a seat	21 August 2018	25	Online exposure of Pinduoduo’s grocery shopping staff dying suddenly on the way to work	3 January 2021
6	A woman takes a Didi Hitch ride in Yueqing and is killed	25 August 2018	26	A 23-year-old woman in Changsha jumped out of the window of a truck and died	21 February 2021
7	Jiangsu Kunshan BMW man was killed after being slashed	28 August 2018	27	21 killed in mountainous marathon accident in Gansu province	22 May 2021
8	Wanzhou bus falling into the river	28 October 2018	28	A gas explosion in Shiyan has killed 25 people	13 June 2021
9	Zhai Tianlin was accused of plagiarism	8 February 2019	29	Extremely heavy rainstorm in Henan	19 July 2021
10	There was an explosion at the Xiangshui chemical plant in Yancheng	21 March 2019	30	Female employees of Alibaba were assaulted	7 August 2021
11	Forest fire broke out in Liangshan	30 March 2019	31	A female passenger is dragged by security guards on the Xi’an subway	30 August 2021
12	A 6.0-magnitude earthquake struck Changning in Yibin	17 June 2019	32	Many places in Northeast China cut off electricity during peak hours	25 September 2021
13	The skeleton of a missing teacher in Hunan province was buried in the playground after 16 years	20 June 2019	33	A mother of eight children in Feng County is mentally unstable and tied to an iron chain	28 January 2022
14	A girl in Hangzhou went missing after being taken by two tenants	9 July 2019	34	MU5735 with 132 people on board crashed in Teng County	21 March 2022
15	Super Typhoon Lekima made landfall	8 August 2019	35	A building collapsed in Changsha	29 April 2022
16	Wuhan and other places have been hit by pneumonia caused by the novel coronavirus	30 December 2019	36	D2809 hit mudslide derailment at Rongjiang Station	4 June 2022
17	A woman drove a Benz into Forbidden City	17 January 2020	37	A group of men beat up a girl at a barbecue restaurant in Tangshan	10 June 2022
18	The use of materials by the Hubei Red Cross Society has been questioned	30 January 2020	38	27 killed and 20 injured after a passenger bus overturned at high speed in Guizhou province	18 September 2022
19	Dr. Li Wenliang, the whistleblower of the epidemic, has died of COVID-19	6 February 2020	39	2 dead and 3 injured after a Tesla loses control in Chaozhou	13 November 2022
20	A designated quarantine hotel collapsed in Quanzhou	7 March 2020	40	10 dead in a high-rise residential building fire in Urumqi	25 November 2022

**Table 2:** Variable assignment table.

Variables	Coding Judgment	Assign	Instructions
Sense of relative deprivation	There is a sense of relative deprivation	1	Condition variable
	There is no sense of relative deprivation	0	
Group identity	There is no group identity	1	Condition variable
	There is group identity	0	
Intergroup threat	There is intergroup threat	1	Condition variable
	There is no intergroup threat	0	
Speed of government response	The speed of the official response	days	Condition variable
The guidance of opinion leaders	The product of the influence of the opinion leaders participating in the discussion and the number of posts	Degree of participation	Condition variable
The help of the media	The number of media participating in the topic discussion	number	Condition variable
Rumors	Rumors arise	1	Condition variable
	No rumors	0	
Irritant event	Irritant events occurred	1	Condition variable
	No irritant events occurred	0	
Positive group emotions	Degree of positive emotion	The proportion of positive emotion	Result variable
Negative group emotions	Degree of negative emotion	The proportion of negative emotion	Result variable

**Table 3:** Data calibration rules.

Category	Element Name	Calibrated Anchor Point		
		Full Affiliated Points	Intersection Points	Completely Unaffiliated Points
Condition variable	Sense of relative deprivation	1	-	0
	Group identity	1	-	0
	Intergroup threat	1	-	0
	Speed of government response	42.500	2.000	1.000
	The guidance of opinion leaders	111.450	40.500	13.950
	The help of the media	471.000	183.500	80.500
	Rumors	1	-	0
	Irritant event	1	-	0
Result variable	Positive group emotions	1	-	0
	Negative group emotions	1	-	0

**Table 4:** Results of necessity inspection.

Antecedent Condition	Outcome Variable: Positive Group Emotion		Outcome Variable: Negative Group Emotion	
	Consistency	Coverage	Consistency	Coverage
Sense of relative deprivation	0.7350	0.4398	0.7230	0.5031
~Sense of relative deprivation	0.2650	0.4180	0.2770	0.5082
Group identity	0.0964	0.4181	0.1007	0.5081
~Group identity	0.9036	0.4356	0.8993	0.5041
Intergroup threat	0.6107	0.7382	0.4842	0.6806
~Intergroup threat	0.7880	0.5333	0.8437	0.6640
Speed of government response	0.6585	0.6582	0.6043	0.7025
~Speed of government response	0.7997	0.6129	0.7720	0.6881
The guidance of opinion leaders	0.6859	0.6287	0.6981	0.7441
~The guidance of opinion leaders	0.7798	0.6423	0.7038	0.6741
The help of the media	0.8848	0.4387	0.8747	0.5043
~The help of the media	0.1152	0.3998	0.1253	0.5057
Rumors	0.6881	0.4118	0.7424	0.5166
~Rumors	0.3119	0.4920	0.2576	0.4725
Irritant event	0.6242	0.4012	0.7328	0.5477
~Irritant event	0.3758	0.5016	0.2672	0.4147

**Table 5:** Conditional variable configuration intermediate solution of positive group emotions.

	Raw Coverage	Unique Coverage	Consistency
RD*~GI*TG*~OL*HM*IT*~IE	0.1198	0.1198	0.9984
~RD*GI*TG*~OL*~HM*~IT*~RC*~IE	0.0289	0.0289	1
RD*~GI*TG*~OL*~HM*IT*~RC*IE	0.0859	0.0859	0.9935
~RD*~GI*TG*OL*HM*IT*~RC*IE	0.0375	0.0375	1
Solution coverage	0.2720		
Solution consistency	0.9972		

Note: * indicates a group relationship between variables.

**Table 6:** Configuration analysis of positive group emotions.

Conditional Configuration	H1a	H1b	H1c	H1d
Sense of relative deprivation	●	ⓧ	·	ⓧ
Group identity	⊗	·	⊗	⊗
Intergroup threat	·	⊗	·	·
Speed of government response	·	●	●	●
The guidance of opinion leaders	ⓧ	⊗	ⓧ	·
The help of the media	·	⊗	⊗	·
Rumors		ⓧ	ⓧ	ⓧ
Irritant event	ⓧ	⊗	·	·
Consistency	0.9984	1	0.9935	1
Original coverage	0.1198	0.0289	0.0859	0.0375
Unique coverage	0.1198	0.0289	0.0859	0.0375
Consistency of solution	0.9972			
The coverage of the solution	0.2720			

Note: ● indicates that core condition exists, · indicates that edge condition exists, ⓧ indicates that core condition is missing, ⊗ indicates that edge condition is missing, and blank indicates that condition is optional.

**Table 7:** Conditional variable configuration intermediate solution of negative group emotions.

	Raw Coverage	Unique Coverage	Consistency
RD*~GI*~TG*~OL*~HM*IT*IE	0.3028	0.0703	0.9593
~RD*GI*~TG*~OL*HM*IT*RC	0.0714	0.0714	0.8791
RD*~GI*~TG*~OL*IT*RC*IE	0.2427	0.0449	0.8608
~RD*~GI*TG*OL*HM*RC*IE	0.0551	0.0551	1
~RD*~GI*~TG*~OL*HM*IT*~RC*~IE	0.0253	0.0253	1
RD*~GI*TG*~OL*~HM*~IT*RC*IE	0.0389	0.0389	0.9234
RD*~GI*TG*OL*~HM*IT*RC*~IE	0.0491	0.0491	1
RD*~GI*~TG*OL*HM*IT*~RC*IE	0.0768	0.0422	0.9572
Solution coverage	0.6295		
Solution consistency	0.9133		

Note: * indicates a group relationship between variables.

**Table 8:** Configuration analysis of negative group emotions.

Conditional Configuration	H2a	H2b	H2c	H2d	H2e	H2f	H2g
Sense of relative deprivation	·	ⓧ	·	ⓧ	⊗	·	·
Group identity	⊗	·	⊗	⊗	⊗	ⓧ	⊗
Intergroup threat	·	·	·		●	ⓧ	·
Speed of government response	ⓧ	⊗	ⓧ	·	ⓧ	·	●
The guidance of opinion leaders	ⓧ	⊗	ⓧ	·	⊗	⊗	●
The help of the media	⊗	●		●	·	ⓧ	⊗
Rumors		●	·	●	ⓧ	·	·
Irritant event	·		·	·	⊗	·	ⓧ
Consistency	0.9593	0.8791	0.8608	1	1	0.9234	1
Original coverage	0.3028	0.0714	0.2427	0.0551	0.0253	0.0389	0.0491
Unique coverage	0.0703	0.0714	0.0449	0.0551	0.0253	0.0389	0.0491
Consistency of solution	0.9133						
The coverage of the solution	0.6295						

Note: ● indicates that core condition exists, · indicates that edge condition exists, ⓧ indicates that core condition is missing, ⊗ indicates that edge condition is missing, and blank indicates that condition is optional.

## Data Availability

Dataset available on request from the authors.
